# Multiple Endoscopic Access Lumbar Interbody Fusion (MALIF): A New Technique for Unilateral Biportal Endoscopic Lumbar Interbody Fusion Using Monoportal Endoscopic Spine Surgery Techniques

**DOI:** 10.7759/cureus.74942

**Published:** 2024-12-01

**Authors:** Shigeto Hiratsuka, Katsuyuki Sasaki, Nodoka Manabe, Takeshi Kaneko

**Affiliations:** 1 Orthopaedics, Wajo-kai Sapporo Hospital, Sapporo, JPN; 2 Orthopaedics, Kanto Rosai Hospital, Tokyo, JPN; 3 Spine Surgery, East Maebashi Orthopaedic Hospital, Maebashi, JPN; 4 Spine Surgery, Inanami Spine and Joint Hospital, Tokyo, JPN

**Keywords:** assisted full-endoscopic spine surgery, endoscopic lumbar interbody fusion, full-endoscopic spine surgery, unilateral biportal endoscopic lumbar interbody fusion, unilateral biportal endoscopy (ube)

## Abstract

This study aims to present a new endoscopic lumbar interbody fusion technique, multiple endoscopic access lumbar interbody fusion (MALIF), which applies the assisted full-endoscopic spine surgery (AFESS) technique to unilateral biportal endoscopic lumbar interbody fusion (UBE-LIF). AFESS represents an advancement over UBE by utilizing a monoportal scope in the camera portal during biportal endoscopic spine surgery, which enables greater stability, controlled irrigation for muscle preservation, and improved protection of neural structures. We performed MALIF on 15 consecutive patients with symptomatic lumbar spondylolisthesis resistant to conservative treatment and showing radiographic evidence of instability. The technique was performed through a unilateral percutaneous pedicle screw (PPS) incision, minimally exposing the lateral facet joint, followed by superior articular process (SAP) resection and interbody cage placement for indirect decompression and fusion. The mean operative time was 97.4 ± 17.5 minutes (range: 70-129 minutes), with minimal blood loss (mean: 24.3 ± 13.8 ml) and short hospital stay (mean: 15.0 ± 3.5 days). All patients showed symptomatic improvement without major complications, except for one case of transient dysesthesia that resolved within two weeks. MALIF offers three key advantages: (1) multi-angle endoscopic access to the surgical site, (2) precise endoscopic discectomy, and (3) enhanced safety through monoportal sleeve protection of the exiting nerve root. These initial results suggest that MALIF is a promising minimally invasive surgical option for lumbar interbody fusion.

## Introduction

Minimally invasive spine surgery has evolved significantly over recent years, with the unilateral biportal endoscopic (UBE) technique demonstrating favorable outcomes in both decompression and fusion surgeries [1-5]. The UBE technique, which utilizes two separate portals for observation and operation, has shown success in lumbar interbody fusion (UBE-LIF) by maintaining adequate surgical visualization with minimal tissue damage [6-15]. Recently, Kaneko et al. made a significant advancement by developing the assisted full-endoscopic spine surgery (AFESS) technique, which modifies the traditional UBE approach by incorporating a monoportal scope instead of an arthroscope [16]. AFESS offers several distinct advantages over conventional UBE, including enhanced camera stability through bone contact of the monoportal sleeve, controlled saline irrigation, and improved protection of neural structures using the monoportal sleeve as a protective barrier. Building upon these advantages, we adapted the AFESS technique to monoportal endoscopic lumbar interbody fusion surgery. Traditional UBE-LIF's fixed camera and working portal positions limit surgical maneuverability and make portal switching challenging. To address these limitations, we developed multiple endoscopic access lumbar interbody fusion (MALIF), which combines the advantages of AFESS with advanced monoportal surgical technology. This innovative approach enables multi-directional access and facilitates endoscopic view switching between portals for effective surgical manipulation. To ensure the safe implementation of this new surgical technique, we conducted meticulous discussions and simulations prior to its adoption, thereby establishing a standardized surgical procedure. These discussions were conducted via remote conferencing, as the members of our team are based at different institutions. The purpose of this original article is to describe the MALIF technique using AFESS for the treatment of lumbar degenerative spondylolisthesis and to present our initial clinical experience with 15 consecutive cases. We aim to demonstrate how this new technique overcomes the limitations of traditional approaches while maintaining the benefits of minimally invasive surgery.

## Materials and methods

Patient population

Between January and July 2024, 15 consecutive patients (12 females, 3 males; mean age 73.4 ± 4.8 years, range 67-83) with symptomatic lumbar spondylolisthesis underwent L4/5 MALIF at Wajo-kai Sapporo Hospital, Sapporo, Japan. All patients showed persistent symptoms despite conservative treatment for at least 12 weeks. The inclusion criteria were as follows: (1) single-level lumbar spondylolisthesis with lumbar instability at L4/5; (2) degenerative spondylolisthesis with Meyerding grade 1 or 2; (3) follow-up periods more than three months after the surgery. The exclusion criteria were as follows: (1) degenerative spondylolisthesis with Meyerding grade > 3; (2) history of lumbar surgery; (3) history of spine tumor or infection in the lumbar spine; (4) the requirement for direct decompression; (5) multi-level lesions. Lumbar instability was defined as the slippage > 3 mm, segmental angle change > 10° on flexion-extension observed in lateral X-rays.

Clinical evaluation

Operative time, intraoperative blood loss, and length of hospital stay were recorded. Surgery-related complications, including nerve root injury, dural tear, epidural hematoma, and surgical site infection, were also assessed. Clinical outcomes were evaluated using the Oswestry Disability Index (ODI) and the visual analog scale (VAS) for back pain and leg pain. The preoperative, one-month postoperative, and three-month operative ODI and VAS were compared. Statistical analysis was performed using a nonparametric test for continuous variables. A p-value of < 0.05 was considered statistically significant.

A representative case

**Figure 1:**
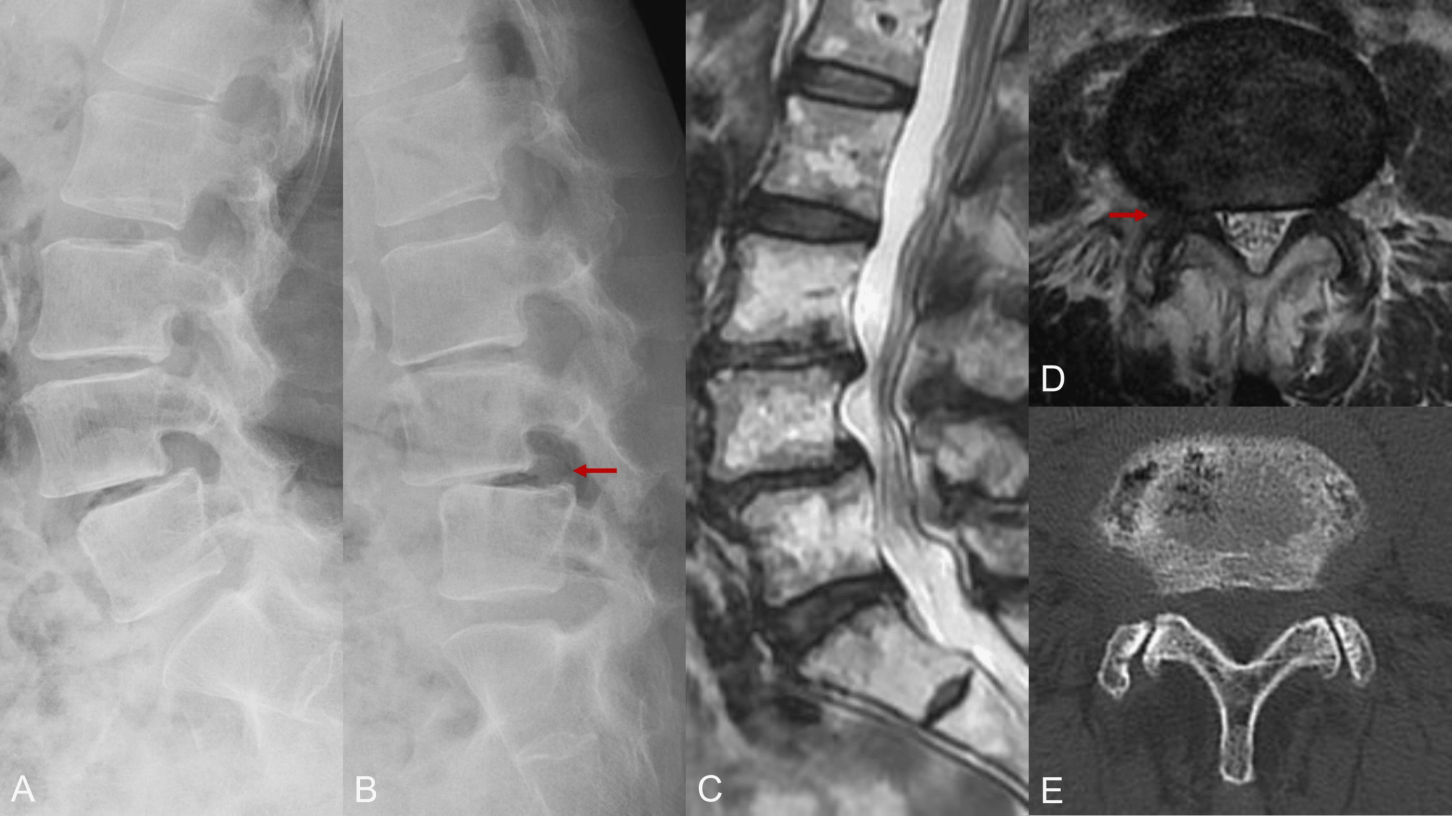
Preoperative imaging (A, B) Plain X-ray showed L4 spondylolisthesis with instability (arrow); (C, D) Sagittal and axial MRI showed lumbar canal stenosis at the L4/5 level (arrow); (E) Axial CT scans at the L4/5 level

Among these cases, we present a representative case of a 77-year-old woman who presented with low back pain and right leg pain. Imaging studies revealed Meyerding grade 2 lumbar spondylolisthesis at L4 with dynamic instability on flexion radiographs (Figures 1A, 1B), and MRI demonstrated spinal canal stenosis at L4/5 (Figures 1C, 1D). After conservative treatment, including pharmacotherapy and physical therapy, proved ineffective, L4/5 MALIF with percutaneous pedicle screw (PPS) fixation was planned.

Surgical technique

The procedure is performed under general anesthesia with the patient in the prone position. Preoperative planning begins with CT-based measurements of PPS entry points from the midline. Under lateral fluoroscopic guidance, PPS trajectories are marked, with the cranial PPS incision aligned with the L4 trajectory and the caudal PPS positioned at the L5 upper endplate. Initial stabilization is achieved by inserting a contralateral PPS and connecting the rod to correct spondylolisthesis and expand the disc space. Subsequently, two 1-cm portals are created on the symptomatic side for the endoscopic procedure.

**Figure 2:**
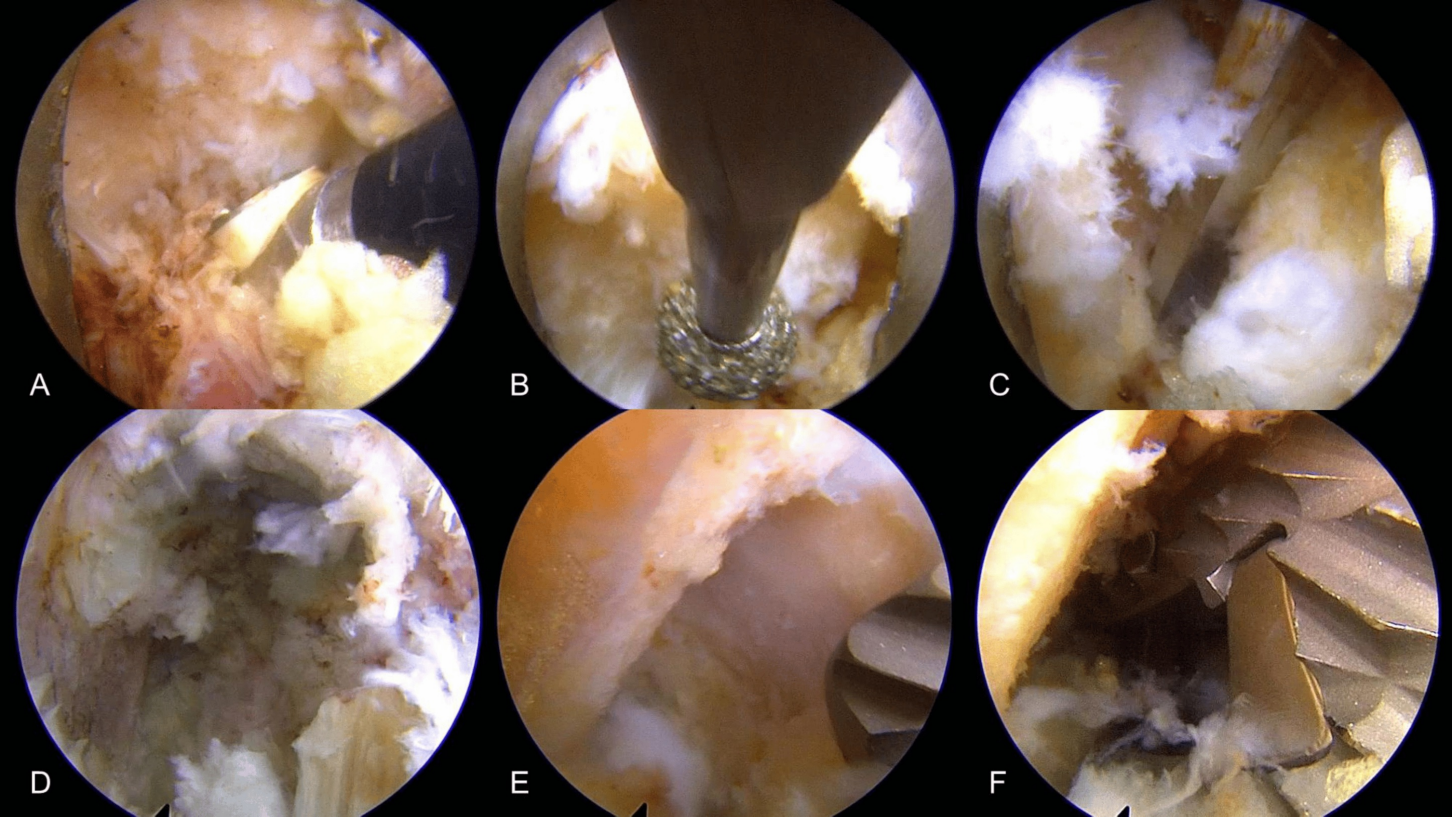
Operative procedure (A) The lateral aspect of the right L4/5 facet joint was exposed by plasma-based radiofrequency (PRF); (B) The base of the L5 superior articular process (SAP) to create a line for osteotomy was drilled while protecting the exiting nerve root (ENR) with a FESS sleeve; (C) The SAP was resected with a chisel; (D) Endoscope inserted into the intervertebral disc space after removing the disc; (E) A groove was created in the L4 inferior articular process (IAP) for guiding the cage into the intervertebral disc space and named the facet slider technique; (F) A cage was inserted along the IAP.

**Figure 3:**
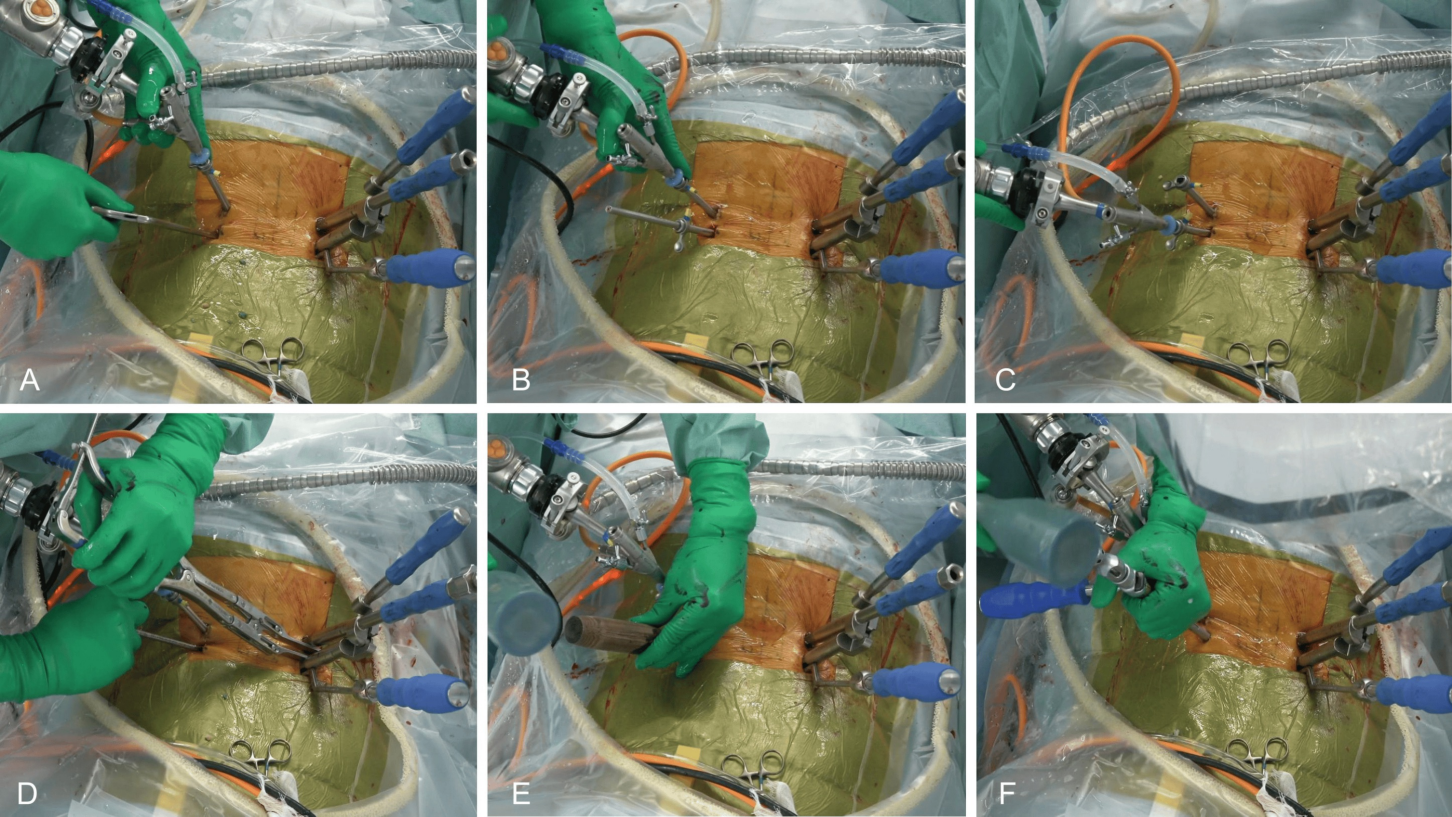
External view of the surgical procedure (A) The assisted full-endoscopic spine surgery (AFESS) technique was done from the unilateral biportal; (B, C) Multiple endoscopic access was performed by switching between the camera portal and the working portal; (D) Distraction between PPSs to correct slippage and expand the intervertebral space; (E, F) Bone resection with a chisel and cage insertion are performed with both hands using the Lock-Arm® endoscope holder.

The endoscopic procedure begins with initial exposure, where the monoportal scope (Elliquence LLC, Baldwin, NY, USA) is placed through the caudal portal and working instruments through the cranial portal, utilizing the AFESS technique to expose the L5 superior articular process (SAP) (Figures 2A, 3A). Bone work and disc preparation follow, involving SAP base drilling (3.5 mm Primado2 Long, NSK-Nakanishi Japan, Tokyo, Japan) and facet joint exposure (Figure 2B). The SAP is then resected using a chisel (Figures 2C, 3E), with the exiting nerve root (ENR) protected by the monoportal sleeve during disc space penetration with a pencil dilator. For discectomy and cage placement, direct endoscopic discectomy is performed through the monoportal sleeve, followed by disc space preparation and sizing (Figure 2D). A local bone graft is placed, and the facet slider technique is employed for cage guidance (Figure 2E). An expandable TLIF spacer (ALTERA®, Globus Medical, Inc., Audubon, PA) is then inserted (Figures 2F, 3F). The procedure is completed with hemostasis confirmation, ipsilateral PPS insertion, and closure without drainage. Figure 3 shows the external view of the surgical procedure.

## Results

**Figure 4:**
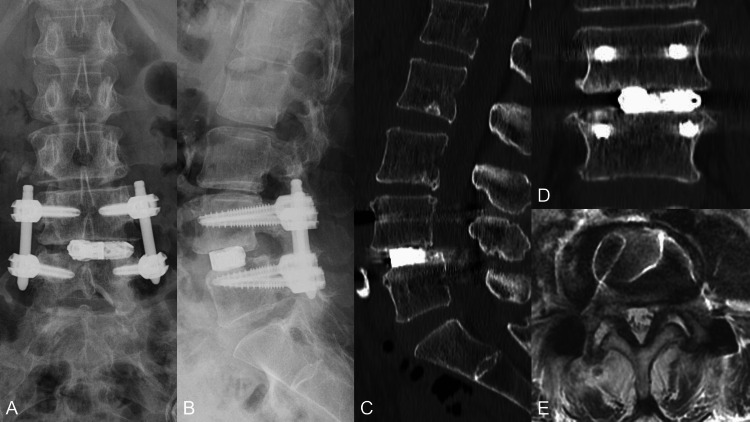
Postoperative imaging (A, B) Plain X-ray showed L4 spondylolisthesis was reduced; (C, D) Sagittal and axial CT scans showed that the cage was in sufficient contact with the L4/5 vertebral endplate; (E) Axial MRI at the L4/5 level showed the spinal canal area was expanded.

In our series of 15 patients, the mean operative time was 97.4 ± 17.5 minutes (range: 70-129 minutes), with an average blood loss of 24.3 ± 13.8 ml (range: 0-60 ml) and mean length of hospital stay of 15.0 ± 3.5 days (range: 7-20 days) (Table 1). In our representative case, the surgery lasted 80 minutes with minimal blood loss. Postoperative imaging showed successful correction of spondylolisthesis and indirect decompression of the spinal canal in all cases (Figures 4A-4E). All patients demonstrated symptomatic improvement, with only one case of transient dysesthesia that resolved within two weeks. At the three-month follow-up, all patients showed significant improvement in visual analog scale (VAS) scores for back and leg pain, as well as ODI scores (Table 2), with no major complications such as nerve root injury, dural tear, epidural hematoma, or surgical site infection. External fixation was maintained for three months postoperatively in all cases, and no findings of apparent nonunion were observed at that time.

**Table 1:** Characteristics of patients undergoing L4/5 MALIF N/A: not applicable; MALIF: multiple endoscopic access lumbar interbody fusion

Case No.	Age (yrs)	Sex	Side of approach	Number of cages	Op time (mins)	Blood loss (ml)	Length of hospital stay (days)	Complications
1	67	F	L	1	129	50	15	N/A
2	77	F	L	1	125	30	15	transient dysesthesia
3	76	F	L	1	118	20	17	N/A
4	83	F	L	1	96	30	20	N/A
5	70	M	R	1	113	60	19	N/A
6	82	M	R	1	108	15	13	N/A
7	68	F	L	1	85	30	17	N/A
8	76	F	L	1	100	20	12	N/A
9	73	F	L	1	78	15	17	N/A
10	72	F	R	2	81	20	10	N/A
11	77	F	R	1	80	10	18	N/A
12	71	F	R	1	105	20	7	N/A
13	67	F	L	1	70	10	17	N/A
14	74	M	R	2	87	10	15	N/A
15	69	F	L	2	85	25	10	N/A

**Table 2:** Preoperative, one-month postoperative, and three-month postoperative outcomes of L4/5 MALIF ODI: Oswestry Disability Index; VAS: visual analog scale *: Significantly different from the operative value (p < 0.05).

Assessment	Preoperative	One-month postoperative	Three-month postoperative
ODI	55.5 ± 13.8	34.7 ± 14.6*	26.7 ± 9.3*
VAS of back pain	7.3 ± 2.1	2.5 ± 1.6*	2.1 ± 1.9*
VAS of leg pain	7.2 ± 2.1	2.8 ± 2.0*	1.8 ± 1.5*

## Discussion

Minimally invasive lumbar interbody fusion using spinal endoscopy has evolved significantly, with various techniques, such as UBE-LIF, percutaneous endoscopic transforaminal lumbar interbody fusion (PETLIF), and full-endoscopic trans-Kambin triangle interbody fusion (fullendo-KLIF), being developed for symptomatic lumbar degenerative diseases with instability [17-19]. Building upon these advances, we have developed MALIF by applying the two-portal AFESS technique to monoportal endoscopic lumbar interbody fusion. Our initial series of 15 cases demonstrates that this novel technique maintains the minimally invasive nature of UBE-LIF while offering several unique advantages. Based on our clinical experience, we identified three distinctive advantages of MALIF beyond the conventional benefits of AFESS.

Multiple endoscopic access

First, MALIF utilizes four skin incisions with a PPS approach, enabling multi-directional surgical access. This represents a significant advancement over traditional two-portal techniques by providing enhanced surgical manipulation through both working portal and monoportal scope, improved visualization through flexible camera positioning, and reduced dependence on ambidextrous skills compared to UBE-LIF. Additionally, it offers the potential for a bilateral approach when necessary. Our series demonstrated that this multiple-access approach contributed to consistent surgical times (mean 97.4 ± 17.5 minutes) and minimal blood loss (mean 24.3 ± 13.8 ml).

Precise endoscopic discectomy

Second, the technique offers several advantages for disc space preparation through direct endoscopic discectomy. Direct visualization of the disc space through monoportal scope insertion enables real-time confirmation of discectomy adequacy while continuous irrigation enhances disc material removal and potentially reduces postoperative infection risk. These features addressed common challenges in endoscopic fusion procedures, potentially contributing to no implant loosening observed in all cases by three months postoperatively.

Monoportal sleeve protection of the ENR

Third, MALIF provides superior protection of the ENR through direct ENR visualization during all critical steps, with the monoportal sleeve serving as a protective barrier during safer cage insertion through Kambin's triangle. This protection system likely contributed to our low complication rate, with only one case of transient dysesthesia among 15 patients.

Technical innovations to enhance the efficacy of MALIF

Our experience has led to several technical refinements that enhance MALIF's effectiveness. The indirect decompression strategy, including preservation of the inferior articular process (IAP), use of expandable TLIF spacers, and implementation of the facet slider technique, resulted in successful indirect decompression in all cases, as confirmed by postoperative imaging. Furthermore, technological enhancements such as the introduction of the Lock-Arm® (System JP Inc., Shizuoka, Japan) flexible endoscope holder enabled true two-handed surgical technique and stable endoscopic visualization throughout the procedure (Figures 3E, 3F).

Limitations and future directions

The present study has several limitations that need to be acknowledged. The technique requires a significant learning curve and technical expertise, which may influence its widespread adoption. It is appropriate to first undergo training in monoportal endoscopic spine surgery and AFESS through workshops and cadaveric training, introduce these techniques into clinical practice, and gain proficiency in endoscopic spine surgery. Similarly, we implemented MALIF after establishing expertise in AFESS techniques. Additionally, the requirement for specialized equipment may limit its implementation in some facilities. MALIF can be performed using conventional spine surgical instruments; however, a full-endoscopic spine surgery setup and devices such as plasma-based radiofrequency and high-frequency bipolar for hemostasis during endoscopic procedures are essential, which incur medical costs. Our current series is limited by its small sample size and relatively short follow-up period, which constrains our ability to evaluate long-term outcomes and potential complications.

Potential contraindications of MALIF must be carefully considered. Since MALIF improves neurological dysfunction through indirect decompression, direct decompression should be considered in cases of severe spinal stenosis with symptoms at rest or ossification of the ligaments. Additionally, when the traversing nerve root is located laterally to the IAP, there is a risk of injury during cage insertion using the facet slider technique with SAP resection. Therefore, preoperative CT or MRI imaging should be thoroughly reviewed to assess the course of the nerve roots and the presence of any anomalies.

Several technical developments are necessary to further optimize the MALIF technique. Refinement of surgical instruments and the development of specialized navigation systems could improve surgical precision and reduce the technical demands of the procedure. The potential extension to multiple-level procedures and integration with robotics technology represents exciting future directions for this technique. The successful implementation of the Lock-Arm® endoscope holder in our series demonstrates how technological innovations can enhance procedural efficiency and safety, setting a precedent for future developments in this field.

## Conclusions

This technical report introduces MALIF, a novel monoportal endoscopic lumbar interbody fusion technique that successfully incorporates the AFESS technique for treating degenerative lumbar spondylolisthesis. Our initial series of 15 consecutive cases demonstrates that MALIF not only preserves the established benefits of AFESS but also offers three significant advantages: multidirectional endoscopic access, direct intradiscal visualization for precise discectomy, and enhanced nerve root protection using the monoportal sleeve. The technique achieved consistent surgical times, minimal blood loss, and favorable clinical outcomes with a low complication rate. All patients showed symptomatic improvement and no implant loosening by three months postoperatively. While further investigation with larger cohorts and longer follow-up is needed, MALIF represents an effective evolution in minimally invasive surgery, offering a safe solution for appropriately selected patients with lumbar spondylolisthesis.
